# Dietary Choline and Betaine and Risk of CVD: A Systematic Review and Meta-Analysis of Prospective Studies

**DOI:** 10.3390/nu9070711

**Published:** 2017-07-07

**Authors:** Katie A. Meyer, Jonathan W. Shea

**Affiliations:** 1 Department of Nutrition, Gillings School of Global Public Health, University of North Carolina at Chapel Hill, Chapel Hill, NC 27599, USA; 2 Nutrition Research Institute, University of North Carolina at Chapel Hill, Kannapolis, NC 28081, USA; jon_shea@unc.edu

**Keywords:** choline, betaine, cardiovascular disease, epidemiology, meta-analysis, systematic review

## Abstract

Studies implicate choline and betaine metabolite trimethylamine N-oxide (TMAO) in cardiovascular disease (CVD). We conducted a systematic review and random-effects meta-analysis to quantify a summary estimated effect of dietary choline and betaine on hard CVD outcomes (incidence and mortality). Eligible studies were prospective studies in adults with comprehensive diet assessment and follow-up for hard CVD endpoints. We identified six studies that met our criteria, comprising 18,076 incident CVD events, 5343 CVD deaths, and 184,010 total participants. In random effects meta-analysis, incident CVD was not associated with choline (relative risk (RR): 1.00; 95% CI: 0.98, 1.02) or betaine (RR: 0.99; 95% CI: 0.98, 1.01) intake. Results did not vary by study outcome (incident coronary heart disease, stroke, total CVD) and there was no evidence for heterogeneity among studies. Only two studies provided data on phosphatidylcholine and CVD mortality. Random effects meta-analysis did not support an association between choline and CVD mortality (RR: 1.09, 95% CI: 0.89, 1.35), but one study supported a positive association and there was significant heterogeneity (*I*^2^ = 84%, *p*-value < 0.001). Our findings do not support an association between dietary choline/betaine with incident CVD, but call for further research into choline and CVD mortality.

## 1. Introduction

A recent body of literature implicates dietary choline and betaine metabolite trimethylamine N-oxide (TMAO) in cardiovascular disease (CVD) risk [1,2,3,4,5,6,7,8]. These findings raise questions about the role of choline and betaine consumption in CVD risk. Food sources of choline and betaine are diverse, including foods that have been postulated to positively and negatively impact CVD—such as red meat, eggs, fish, green vegetables, and whole grain [9]. In addition, non-dietary features of TMAO production and excretion may contribute to substantial individual variability in circulating TMAO [2,10,11,12].

To better understand the role of dietary choline and betaine in CVD risk, we systematically reviewed the literature for studies of dietary choline or betaine with respect to CVD incidence or mortality, and used random-effects meta-analysis to generate summary relative risks. We restricted our analysis to prospective, population-based studies of adults with comprehensive dietary assessment and long-term follow-up ascertainment of hard CVD endpoints. We hypothesize that dietary choline and betaine are not predictive of CVD events. Our investigation has public health implications and addresses the extent to which associations between TMAO and CVD impact dietary advice related to consumption of TMAO precursors choline and betaine.

## 2. Materials and Methods

Through all stages of the study, we followed the Preferred Reporting Items for Systematic Reviews and Meta-Analyses (PRISMA) [13] and Meta-analysis of Observational Studies in Epidemiology (MOOSE) guidelines [14].

### 2.1. Data Sources

We completed systemic searches in three databases (PubMed, EMBASE, and Scopus) for manuscripts reporting results from prospective cohort studies of dietary consumption of choline or betaine with respect to CVD incidence or mortality. Publications were included from the earliest database indexing to 1 April 2017. Search terms included choline, betaine, cardiovascular disease, heart disease, coronary heart disease, coronary artery, peripheral artery, sudden death, myocardial infarction, heart attack, cerebrovascular disease, and stroke (see Appendix A for search details). We additionally conducted hand searches based on the reference lists of eligible publications.

### 2.2. Study Selection

One investigator reviewed all titles and abstracts for potentially eligible articles. Full text of potentially eligible publications were reviewed by both investigators, with instances of disagreement decided by consensus. Eligible studies were publications that presented multivariable-adjusted effect estimates and a measure of uncertainty (e.g., 95% CI) for the association between choline or betaine and CVD incidence or mortality from prospective adult (aged 18+ years) cohorts. We excluded editorials, letters, conference abstracts, and review articles. Studies were excluded if they were not prospective (e.g., cross-sectional); if they were in population subgroups with major comorbidity, such as chronic kidney disease; or if outcomes included only biomarkers or other soft endpoints. Studies were required to derive choline and betaine consumption from a full dietary assessment.

### 2.3. Data Abstraction

From each eligible publication, we extracted study data, including first author; publication year; location of study; study design; population ages, races, and gender; sample size; years of study (baseline to complete follow-up); dietary assessment method; study outcome definition and assessment method; analytic method; covariates included in multivariable-adjusted analysis; multivariable-adjusted effect estimates and 95% confidence intervals (or other measure of uncertainty). For studies with multiple exposure categories (e.g., quartiles), we extracted category-specific nutrient consumption (median), number of events, person-years of follow-up, and effect estimates (and 95% CI).

We estimated necessary data if they were not reported or available from the corresponding author, including median nutrient intake, person-years of follow-up, and the number of events. We estimated category-specific median nutrient intake as category midpoints; if the highest category was open-ended, we assumed the same range as the adjacent category. We estimated category-specific person-years by multiplying the number of participants in the category by the overall mean (or median) person-years. We estimated the category-specific number of events from category-specific effect estimates and category-specific person-years of follow-up.

### 2.4. Quality Scoring

We used the Newcastle-Ottawa Scale (NOS) for cohort studies to assess studies with respect to quality [15,16]. We assigned one point for each of the eight quality criteria that the study met, with the sum of scores across criteria reflecting overall study quality. The NOS comprises items related to the representative selection of study participants, ascertainment of exposure and outcome, control for confounding, and loss to follow-up. We considered scores of 0–2 to be low quality, 3–5 to be moderate quality, and 6–8 to be high quality. In addition, we noted the extent to which necessary category-specific data elements were estimated, including median nutrient intakes, person-years of follow-up, and the number of events (as described above).

### 2.5. Statistical Analysis

We estimated linear dose–response across nutrient categories using generalized least-squares models [17], with linear regression estimates standardized to reflect daily intake of 100 mg choline or 35 mg betaine, corresponding to levels in commonly-consumed foods [9]. For studies that reported results only by study subgroups (e.g., gender), we pooled subgroup-specific estimates and variances using fixed effect meta-analysis with inverse-variance weighting. We then pooled study-specific estimates to derive an overall estimate using inverse-variance weighted random-effects meta-analysis [18]. Smaller studies are weighted more heavily in random effects estimation, and we included fixed effects estimation for comparison where there was no evidence for heterogeneity across studies. Separate analyses were conducted for choline and betaine and for incidence and mortality outcome measures. Heterogeneity among studies was quantified by the *I*^2^ statistic [19]. We considered an *I*^2^ statistic greater than 75% as an indication of a high level of heterogeneity. Publication bias was assessed using funnel plots [20] if the number of studies was greater than or equal to ten [21]. If data from the same cohort were reported in more than one publication, we used data from the report with (in order of preference): (1) the larger sample size; (2) longer follow-up; or (3) broader CVD outcome definition. All analyses were completed using Stata 14.0 (Stata Corp, College Station, TX, USA). Statistical significance was defined as a 2-tailed alpha level of 0.05.

## 3. Results

### 3.1. Study Characteristics

From 5398 unique abstracts, we identified 31 publications for full text review, and six eligible manuscripts reporting estimated effects of dietary choline or betaine on cardiovascular disease outcomes (Table 1). All eligible reports were published in English. Five distinct cohorts were included, reflecting three countries (USA, The Netherlands, and Japan) and three race groups (Caucasian, African-American, and Asian). Five of the six studies included results on CVD incidence; two included results on CVD mortality.

Two publications reported results from the Nurses’ Health Study and Health Professionals Follow-up Study [22,23]. In 2014, Bertoia et al. [22] reported on dietary choline and betaine (separately) with respect to incident peripheral vascular disease; in 2016, Zheng et al. [23] reported on the intake of phosphatidylcholine, the major dietary source of choline in the US, with respect to CVD mortality, CVD incidence, and CHD incidence. The Zheng et al. [23] study had a larger sample size, comprising both Nurses’ Health Study and Nurses’ Health Study II cohorts, longer follow-up, and included total CVD and CHD events. Therefore, we included Zheng et al. [23] in analysis of choline, and Bertoia et al. [22] in analysis of betaine.

Study quality was high, with quality scores ranging from 6–8 (on a scale of 8). CVD outcomes were generally ascertained by death certificates, medical record review, or hospital discharge data; where incident outcomes were based on self-report, studies included objective confirmation. In all studies, diet was assessed by food frequency questionnaire (FFQ). All studies included covariate adjustment for socio-demographics, health behaviors, measures of CVD risk, and total energy intake; Millard et al. [24] adjusted for the fewest total covariates, particularly with respect to other aspects of diet.

### 3.2. Choline and Incident CVD

**Figure 1:**
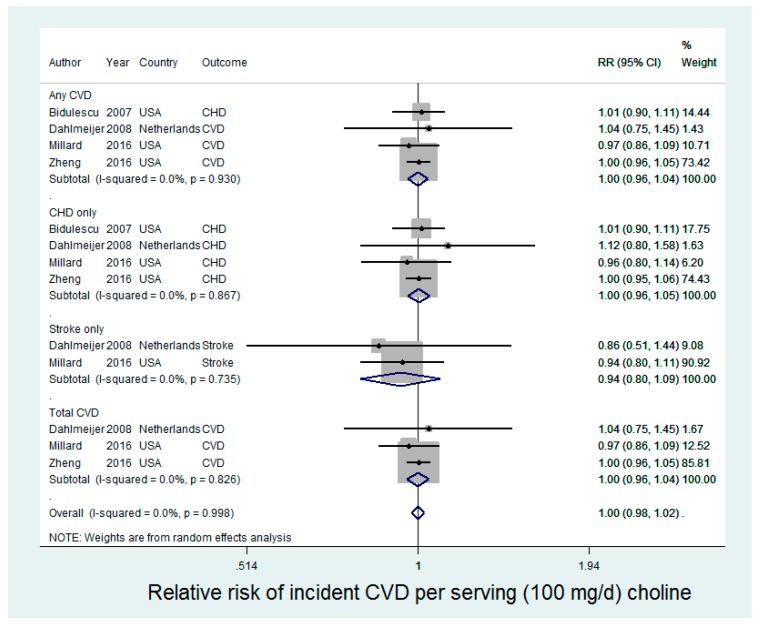
Choline intake and CVD incidence.

Four studies were included in the analysis of choline and incident CVD (CHD, stroke, or total CVD), contributing 17,286 cases of any CVD in 154,931 participants (Figure 1). In random-effects meta-analysis, choline was not associated with any incident CVD (relative risk (RR): 1.00 (95% CI: 0.98, 1.02) for an increase of 100 mg/day choline intake). There was no statistical evidence for heterogeneity (*I*^2^ = 0%, *p*-value = 0.93). Results were similar in analysis for other CVD outcome groups (CHD only, stroke only, and total CVD).

### 3.3. Betaine and Incident CVD

**Figure 2:**
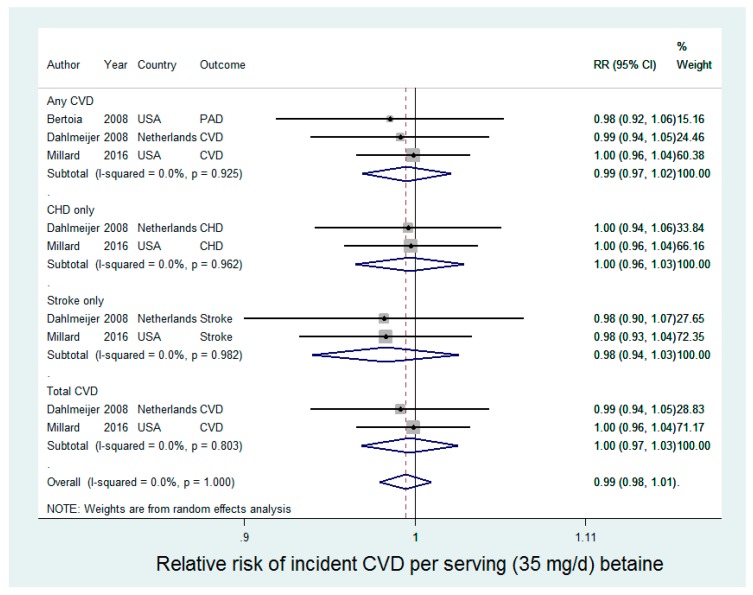
Betaine intake and CVD incidence.

Three studies were included in the analysis of betaine and incident CVD (CHD, stroke, or total CVD), contributing 1660 cases of any CVD in 136,941 participants (Figure 2). Betaine was not associated with any incident CVD [relative risk (RR): 0.99 (95% CI: 0.97, 1.02) for an increase of 35 mg/day choline intake] in random-effects meta-analysis (Figure 2), with no statistical evidence for heterogeneity (*I*^2^ = 0%, *p*-value = 0.93).

### 3.4. Choline and CVD Mortality

**Figure 3:**
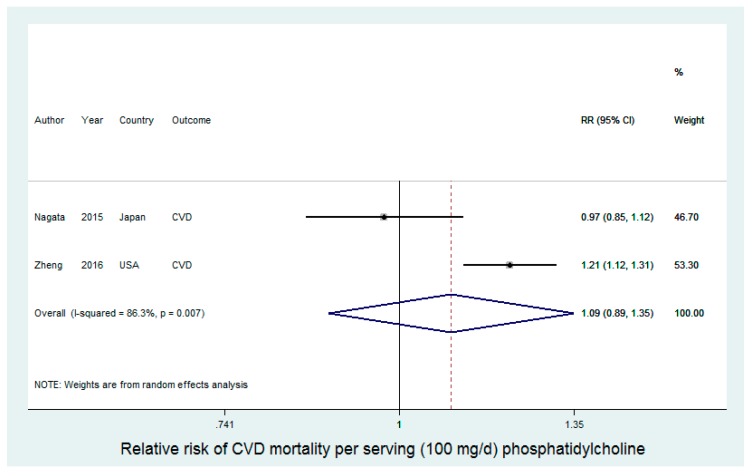
Phosphatidylcholine intake and CVD mortality.

Two studies reported results for phosphatidylcholine with respect to CVD mortality, with a total of 5342 events and 165,215 study participants (Figure 3). There was a high level of heterogeneity between the two studies (*I*^2^ = 86.3%, *p*-value < 0.01). In a Japanese sample, Nagata et al. [27] reported a relative risk for CVD mortality of 0.97 (95% CI: 0.85, 1.12) for a 100 mg/day increase in phosphatidylcholine intake. In contrast, in a U.S. sample, Zheng et al. [23] reported a relative risk for CVD mortality of 1.21 (95% CI: 1.12, 1.31) for a 100 mg/day increase in phosphatidylcholine intake. The summary relative risk from random effects meta-analysis did not support an effect of phosphatidylcholine on CVD mortality (RR = 1.09 (95% CI: 0.89, 1.35)). Nagata et al. [27] additionally conducted analysis of total choline intake and individual choline-containing compounds; we included phosphatidylcholine in the present study for comparability with Zheng et al. [23], who included only phosphatidylcholine. In the analysis of total choline, Nagata et al. reported that they found significant inverse associations for choline (RR = 0.58 (95% CI: 0.36, 0.93)) and betaine (RR = 0.62 (95% CI: 0.39, 0.998)) with CHD death in men, but not in women. However, confidence intervals were wide, the trend was not statistically significant, and a test for interaction by gender was not presented. Zheng et al. [23] also conducted subgroup analysis, and demonstrated that the risk of CVD mortality was significantly stronger (*p*-value interaction: 0.001) among individuals with diabetes, as compared to individuals without diabetes, though phosphatidylcholine was significantly associated with increased CVD mortality in both subgroups (respectively, RR (95% CI: 1.67 (1.26, 2.20) and 1.19 (1.07, 1.31)).

### 3.5. Betaine and CVD Mortality

There was only one study on betaine and CVD mortality (984 events and 29,079 participants). Betaine was not associated with CVD mortality, with a relative risk of 0.98 (95% CI: 0.85, 1.12) for every 35 mg/day increase in betaine intake.

### 3.6. Publication Bias

We did not perform small-study analysis, as the Cochrane Handbook recommendation to restrict such analyses to investigations with at least 10 studies [21].

## 4. Discussion

In this systematic review and meta-analysis of prospective studies, neither dietary choline nor betaine were associated with incident CVD. There were only two studies of choline and CVD mortality, with inconsistent findings. Studies included in the meta-analysis were of high quality and reflected diverse samples, with three countries and three race groups represented. Studies contributed a large number of CVD events and person-time of follow-up, including 18,076 incident CVD events and 5343 CVD deaths from 184,010 total participants. In random-effects meta-analysis, there was no association between either choline or betaine and incident CVD, and no statistical evidence for heterogeneity across studies. There was significant heterogeneity in the two studies of choline and CVD mortality, with a large study supporting a statistically significant positive association with CVD deaths over 32 years of follow-up. In summary, our findings do not support a role for choline or betaine in CVD incidence, but indicate the need for additional research into choline and CVD mortality.

Our study was motivated by the question of whether dietary precursors of choline and betaine metabolite trimethylamine N-oxide (TMAO) are associated with CVD in large-scale prospective cohort studies. There is a growing body of literature implicating choline and betaine metabolite TMAO in CVD risk [1,2,3,4,5,6,7,8]. The generation of TMAO relies on: (1) the consumption of dietary precursors; (2) conversion to TMA by gut microbiota; and (3) oxidation by liver flavin-containing monooxygenases (FMOs) of TMA to TMAO [2,11,12,28]. A genome-wide association study (GWAS) of mouse and human studies indicates that relatively little of the variability in TMAO can be explained by FMO variants and that TMAO variability primarily reflects dietary consumption of precursors and differences in the gut microbiota [10]. In the present study, we specifically focused on the role of dietary precursors choline and betaine in CVD to address the question of how the TMAO-CVD findings may impact dietary recommendations related to the consumption of TMAO precursors.

Our results do not support population benefit from decreasing the intake of choline or betaine as a primary prevention strategy for incident CVD. These results were robust to race and country, which may contribute variability in the consumption of dietary sources of choline and betaine, including foods hypothesized to independently influence CVD risk. In addition, decreasing choline and betaine intake may have adverse health effects, particularly related to methylation, lipid metabolism, and neurotransmitter synthesis [29,30]. Furthermore, in three of the five studies that included total choline, the median intake at even the highest category of consumption did not meet the adequate intake (AI) for prevention of liver damage [31] of 425 mg/day for women and 550 mg/day for men [22,25,26]. Choline intake was higher in the Jackson Heart Study [24], an African-American cohort, and in the Japanese sample [27], but in each study at least half of the participants were below the recommended AI.

In contrast to the consistent findings for incident CVD, findings for choline intake and CVD mortality were divergent. We identified only two studies of CVD mortality that met our study criteria. In one study, Zheng et al. reported a positive association between phosphatidylcholine intake and CVD mortality [23]. Phosphatidylcholine is a major source of dietary choline in the US [32] and accounted for 54% of total choline intake in the Zheng et al. study [23]. In contrast to Zheng et al. [23], Nagata et al. found that phosphatidylcholine intake was not associated with CVD mortality in a Japanese sample [27]. We note that Nagata et al. [27] also reported results for total choline intake and other choline compounds; we elected to use results for phosphatidylcholine for comparability with Zheng et al. [23]

With only two studies, we were not able to determine sources of heterogeneity that may explain differences in findings. Nagata et al. [27] was conducted in Japan, while Zheng et al. [23] was conducted in a relatively homogeneous US population of mostly European-Americans. In addition to racial and country differences, Nagata et al. [27] participants were older, and had a higher prevalence of risk factors at study baseline. In particular, the Japanese sample had a higher baseline prevalence of type 2 diabetes [27] than Zheng et al. [23], which is especially relevant given Zheng et al.’s findings that choline was more strongly associated with CVD deaths among individuals with diabetes [27]. Differential study results also could not be attributed to differences in the level of, or variability in,choline intake: intakes ranged from 138 to 290 in women and 152–321 in men in Nagata et al. [27] and 130–236 in women and 140–261 in men in Zheng et al. [23] The studies differed with respect to CVD causes of death, with stroke more common than CHD in Nagata et al. [27], but neither stroke nor CHD mortality was associated with phosphatidylcholine in Nagata et al. [27] Study samples likely differ in their consumption of food sources of choline, and—although both studies included adjustment for several dietary variables—there is the potential for residual confounding by diet. For example, in the Japanese study, seafood accounted for a much larger percentage of choline intake (15.2% in men and 13.9% in women) [27] as compared to a US sample (5.2%) [33]. Gut microbiota data were not available in either study, and it is possible that differences in the gut microbial community contributes to divergent study findings, perhaps through variable generation of TMAO.

These findings highlight the need for further work to delineate sources of heterogeneity and refine our understanding of a possible role for choline in CVD mortality. Taken together, however, our analysis suggests that choline may—at least in some populations—increase the risk of CVD mortality, while not influencing incident CVD. Indeed, in the same study in which they reported the positive association between phosphatidylcholine and CVD mortality, Zheng et al. found no association between phosphatidylcholine and incident CVD [23]. These findings suggest that choline intake may—perhaps through generation of TMAO—influence the prognosis of CVD, but not the development of CVD. Such a model is not inconsistent with the current literature on TMAO and CVD.

Studies that show a positive association between TMAO and CVD risk have generally been conducted in clinic-based samples at high risk for CVD risk or in disease-based cohorts, such as groups with chronic kidney disease [1,2,3,4,5,6,7,8]. Zheng’s finding of a stronger choline effect on CVD mortality among individuals with diabetes may similarly reflect an etiology dependent on the extent of underlying disease [23]. In further support of this model, plasma TMAO was not associated with the progression of subclinical atherosclerosis in a population-based cohort of healthy individuals [34].

Our analysis has several strengths. We followed standardized protocols for the conduct of systematic reviews and meta-analysis of observational studies and defined precise inclusion criteria to ensure that our analysis included all available high-quality studies. Studies were of high quality, with large samples, covariate adjustment, comprehensive dietary assessment, and long-term follow-up for hard CVD endpoints. Study data came from three countries and three racial groups were represented, which limits confounding by covariates that would differ among samples, such as diet. Given consistent findings for no effect of choline or betaine on incident CVD, it is unlikely that these results were influenced by publication bias. Although results were divergent for phosphatidylcholine and CVD mortality, the positive finding was in the largest study in the entire meta-analysis, arguing against a small-study bias.

Our investigation also has several limitations. Measurement error in diet may have attenuated findings. There is large error in the assessment of both choline and betaine from FFQ, with reliability coefficients of roughly 0.50 [35], though we note similar reliability estimates have been reported for nutrients for which the evidence with respect to CVD risk is more consistent, including dietary fiber, and saturated and polyunsaturated fatty acids [36]. Studies included adjustment for a range of covariates, but the potential for residual confounding remains. There were few eligible studies for inclusion in the analysis, and we lacked power for subgroup analyses that may be informative, such as related to variation in the observation period and participant characteristics of included cohorts. The small number of studies also limited the usefulness of certain statistical tests that are typically included in meta-analyses [21], such as an Egger’s test for small-study bias and accompanying Funnel plots. In part, the small number of studies likely reflects the relatively recent introduction of choline and betaine estimates into nutrient databases of cohort studies [9,32].

The scientific advantages of a systemic review must be considered alongside the possibility that some relevant material does not meet study eligibility criteria. For example, in a cross-sectional study of adults, Detopoulou et al. reported inverse associations between dietary choline and betaine with circulating concentrations of inflammatory markers, including C-reactive protein, interleukin-6, and tumor necrosis factor-alpha [37]. However, in addition to the cross-sectional design and lack of hard endpoints, this study did not include adjustment for dietary covariates or total energy intake.

In conclusion, in this systematic review and meta-analysis of prospective cohort studies, we found no evidence of a positive association between dietary choline or betaine and incident CVD. The summary estimate for dietary choline and CVD mortality was also not statistically significant, but this analysis was based on only two studies; there was appreciable heterogeneity between the studies, with one showing a positive association between phosphatidylcholine and CVD death. These findings have relevance for dietary recommendations, and do not indicate a population benefit to decreasing intake of choline- or betaine-rich foods in the prevention of CVD. Given the paucity of studies on choline intake and CVD mortality, the observed heterogeneity between the two eligible publications, and the growing body of literature supportive of an etiologic role of choline metabolite TMAO in CVD in non-health samples, further work is needed to define the prognostic relevance of choline intake in CVD mortality.

## Figures and Tables

**Table 1:** Characteristics of six prospective cohort studies reporting the association between dietary choline or betaine and cardiovascular disease.

Author (Year)	Country	Study Sample	Study Period	Diet Assess-ment	Exposure Measure	Median Intake per Category (mg/Day)	Outcome Ascertain-ment	Outcome Measure	Sample Size	*N* Events	Total Person-Years	RR (95% CI)	Covariate Adjustment	Quality Score
Bertoia (2014) [22]	United States	Women aged 44–69 at baseline (Nurses’ Health Study); men aged 40–75 at baseline (Health Professionals Follow-up Study)	1990–2010 for women; 1986–2010 for men	FFQ	Total dietary choline (mg) and betaine (mg)	Women: (Choline: 246, 282, 307, 334, 377; Betaine: 67, 85, 101, 120, 159); Men: (Choline: 304, 348, 379, 415, 488; Betaine: 81, 102, 121, 144, 191)	Self-report with medical record adjudication	Peripheral artery disease	116,852 (72,348 women, 44,504 men)	790 (274 in women and 516 in men)	1,302,032 (723,480 women, 578,552 men)	Q5 v Q1: Women: Choline: 1.07 (0.72–1.60); Betaine: 1.02 (0.69–1.52), Men: Choline: 1.24 (0.91–1.68); Betaine: 1.02 (0.77–1.35)	Age, total energy intake, race, smoking, hypertension, high cholesterol, diabetes, family history of MI, BMI, alcohol consumption, physical activity, aspirin use, postmenopausal hormone use (women only).	6
Bidulescu (2007) [25]	United States	Men and women in the biracial (European- and African-American) ARIC cohort, aged 45–64 at baseline	1987–2002	FFQ	Total dietary choline	Choline: 109, 250, 323, 403	Self-report with medical record adjudication; community surveillance of hospital discharge and death certificate data	Coronary heart disease (CHD)	14,430	1072	202,020	Q4 v Q1: Choline: 1.09 (0.79–1.50)	Age, gender, education, total energy intake, dietary folate, methionine and vitamin B6, race, diabetes, field center, menopausal status (women only), dietary cholesterol, dietary intake of saturated fatty acids, animal fat, dietary fiber, and animal protein.	8
Dalmeijer (2008) [26]	Nether-lands	Female participants in a breast cancer screening sub-study of the Prospect-EPIC cohort	1993–1997 (base-line) through 2004	FFQ	Total dietary choline (mg) and betaine (mg)	Choline: 245, 282, 311, 356; Betaine: 162, 214, 257, 322	Electronic hospital discharge database (Dutch Centre for Health Care Information) and death registries	CVD	16,165	717	130,667	Q4 v Q1: Choline: 1.04 (0.71–1.53); Betaine: 0.90 (0.69, 1.17)	Age; physical activity; smoking; diabetes; hypertension; BMI; hypercholesterolemia; systolic blood pressure; intake of total energy, protein, saturated fat, monounsaturated fat, polyunsaturated fat, alcohol, vitamin B2, vitamin B6, vitamin B12, folate, choline (betaine model), betaine (choline model).	7
								CHD	16,165	493	130,667	Choline: 1.28 (0.86–1.91); Betaine: 0.95 (0.72, 1.25)		
								Stroke	16,165	224	130,667	Choline: 0.61 (0.33-1.13); Betaine: 0.83 (0.55, 1.25)		
Millard (2016) [24]	United States	Men and women from the African-American Jackson Heart Study, aged 21–94 at baseline	2000–2004 (base-line) through 2011	FFQ	Total dietary choline (mg) and betaine (mg)	Choline: 125, 239, 332, 730; Betaine: 40.6, 87.4, 128, 478	Self-report; hospital discharge; physician office visit records	CVD	3924	153	35,316	Q4 v Q1: Choline: 0.58 (0.28, 1.20); Betaine: 1.07 (0.66, 1.73)	Age, gender, smoking, systolic blood pressure, antihypertensive medication, fasting plasma glucose, total- to HDL-cholesterol ratio, dietary methionine, total energy intake.	6
								CHD	3924	124	35,316	Choline: 0.66 (0.27, 1.60); Betaine: 1.20 (0.68, 2.11)		
								Stroke	3924	75	35,316	Choline: 0.41 (0.16, 1.09); Betaine: 0.56 (0.28, 1.14)		
Nagata (2015) [27]	Japan	Men and women from the Takayama Study, aged 35+ at baseline	1992–2008	FFQ	Total dietary choline (mg) and betaine (mg)	Choline: Women: 307, 388, 442, 525, Men: 362, 445, 513, 614; Betaine: Women: 170, 239, 288, 377, Men: 208, 287, 350, 463	Death certificates	CHD	29,079 (15,724 women, 13,355 men)	308 (154 women, 154 men)		Q4 v Q1: Women: Choline: 0.80 (0.40, 1.60); Betaine: 0.90 (0.53, 1.51), Men: Choline: 1.08 (0.57, 2.04); Betaine: 0.60 (0.37, 0.97)	Age; BMI; physical activity; smoking; education; marital status; menopausal status (women); vitamin supplement use; aspirin use; history of diabetes or hypertension; intakes of total energy, alcohol, saturated fat, polyunsaturated fat, folate, salt, and fruit.	7
								Stroke	29,079 (15,724 women, 13,355 men)	676 (349 women, 328 men)	410,382 (227,083 women, 183,299 men)	Women: Choline: 1.02 (0.65, 1.60); Betaine: 1.04 (0.72, 1.49), Men: Choline: 0.98 (0.62, 1.55); Betaine: 0.84 (0.59, 1.20)		
Zheng (2016) [23]	United States	80,978 women (NHS), aged 34–59 at baseline; 39,434 men (HPFS), aged 40–75 at baseline	1980–2012 women; 1986–2012 men	FFQ	Dietary phospha-tidyl-choline (mg)	Phosphatidyl-choline: Women: 130, 154,171, 191, 236; Men: 140, 166, 187, 212, 261	Mortality: Death certificates and medical records; Morbidity: Self-report with confirmation by medical record review	CVD, mortality	120,412 (80,978 women, 39,434 men)	4357 (2297 women, 2060 men)	2,828,658 (2,078,089 women, 748,911 men)	Pooled over gender: 1.26 (1.15, 1.39), Women: 1.19 (1.05, 1.35), Men: 1.39 (1.20, 1.61)	Age, BMI, race, marital status, menopausal status and HRT (women), family history of CVD, smoking, alcohol consumption, physical activity, presence of diabetes, hypertension, or hypercholesterolemia, regular aspirin use, dietary energy, *trans* fat, polyunsaturated-to-saturated fat ratio.	7
								CVD, incidence	120,412	15,344	3,199,530	1.00 (0.95, 1.06)		
								CHD, incidence	120,412	11,305	3,201,620	1.01 (0.95, 1.07)		

RR: relative risk; CVD: cardiovascular disease; CVD: coronary heart disease; PAD: peripheral artery disease; FFQ: food frequency questionnaire; BMI: body mass index; ARIC: Atherosclerosis Risk in Communities; EPIC: European Prospective Investigation into Cancer and Nutrition; HRT: hormone replacement therapy; NHS: Nurses’ Health Study; HPSF: Health Professionals Follow-up Study.

## Appendix A. Database Search Strategy

PubMed: (“cardiovascular diseases” [MeSH Terms] OR “heart disease” [all fields] OR “heart diseases” [all fields] OR “myocardial infarction” [all fields] OR “myocardial infarctions” [all fields] OR “heart attack” [all fields] OR “heart attacks” [all fields] OR “carotid artery” [all fields] OR “coronary artery” [all fields] OR “peripheral artery” [all fields] OR “coronary syndrome” [all fields] OR “sudden death” [all fields] OR “sudden deaths” [all fields] OR (“cardiovascular” [All Fields] AND “disease” [All Fields]) OR “cardiovascular disease” [All Fields] OR “cerebrovascular disorders” [mesh terms] OR “stroke” [mesh terms] OR “carotid artery diseases” [mesh terms] OR “stroke” [all fields] OR “cerebrovascular disease” [all fields]) AND (“choline” [mesh terms] OR “choline” [all fields] OR “betaine” [mesh terms] OR “betaine” [all fields])

EMBASE: (“cardiovascular”/exp OR cardiovascular OR coronary OR “stroke”/exp OR cerebrovascular) AND (“choline”/exp OR choline OR “betaine”/exp OR betaine)

Scopus: (cardiovascular OR coronary OR stroke OR cerebrovascular) AND (choline OR betaine).
